# Enhanced episodic specificity and socioemotional content in older adults’ everyday autobiographical thoughts

**DOI:** 10.1073/pnas.2513990123

**Published:** 2026-01-08

**Authors:** Vannia A. Puig Rivera, Eric Andrews, Leelu J. Cervantes, Delaney Freveletti, Matt Huentelman, Matthew D. Grilli, Jessica R. Andrews-Hanna

**Affiliations:** ^a^Psychology Department, The University of Arizona, Tucson AZ 85721; ^b^Applied Psychology Department, Northeastern University, Boston MA 02115; ^c^Division of Early Detection and Prevention, Translational Genomics Research Institute, TGen, Phoenix AZ 85004; ^d^Evelyn F. McKnight Brain Institute, University of Arizona, Tucson AZ 85719; ^e^Cognitive Science Department, The University of Arizona, Tucson AZ 85721

**Keywords:** aging, autobiographical memory, ecological momentary assessment, language analysis, spontaneous thoughts

## Abstract

Cognitive aging research has long observed that older adults’ autobiographical memories and future thoughts, as assessed in laboratory contexts, lack spatiotemporal detail compared to young adults. Does this pattern also hold in everyday contexts? Across two studies, we examined characteristics of autobiographical thinking in real-world settings using ecological momentary assessment (EMA). Study 1 included an adult lifespan sample (N = 3,847). Study 2 (preregistered) included 217 young and older adults whose autobiographical memories were also assessed with a traditional lab-based interview. Contrary to lab-based findings commonly reported in the literature, older adults across both studies reported more episodic specificity in their everyday autobiographical thoughts than younger adults, as well as shifts toward positive and social focus rather than on the self. Linguistic analyses validated self-report data, revealing greater concreteness and perceptual detail with increasing age. Together, these findings highlight discrepancies between measurement approaches in autobiographical thinking in older age, emphasizing the significance of motivational and contextual factors in its study.

Decades of research in cognitive aging suggest that older adults remember their past and future with less detail and specificity than young adults, often providing memories that are more general or semantic in nature (1). These findings, alongside age-related neural alterations in medial temporal and prefrontal regions (2), have contributed to a dominant, decline-oriented view of aging and autobiographical thought. A growing body of work hints that such age effects may partly reflect the social and contextual constraints of laboratory settings (3), which may not fully capture mechanisms that older adults rely on to support memory function in daily life (4). Here, we evaluate the possibility that evaluating naturally unfolding autobiographical thoughts in everyday settings may paint a different picture of cognitive aging. Traditional assessments of autobiographical memory and future thoughts typically involve structured interviews where experimenters prompt participants to share details about autobiographical events (5). In contrast, EMA enables real-time sampling of autobiographical thoughts that arise in more familiar and natural environments, capturing privately experienced thoughts which may differ phenomenologically from that inferred from participants’ shared descriptions in social settings (6). In two studies, we examined everyday autobiographical thinking across the adult lifespan, focusing on characteristics commonly assessed in laboratory settings, such as episodic specificity, affective content, and self/other focus.

## Methods

Study 1 employed an EMA approach using a freely available mobile application developed by our research group, called Mind Window, to examine age differences in subjective (self-report) and objective (linguistic) characteristics of autobiographical thought. Participants received six random “check-in” prompts per day, each prompting a survey (~2 min) about their mood and thoughts just prior to the alert. A more detailed description of study procedures, including survey questions, is described in the Supporting Information.

Study 2 (preregistered) aimed to replicate the age group differences found in Study 1 in a sample of participants who also completed the Autobiographical Interview (5), an established laboratory measure of autobiographical memory commonly showing reduced episodic specificity in older adults (1, 5). Thus, Study 2 allowed us to compare autobiographical thoughts from laboratory and naturalistic settings in participants who completed both measures. The full experimental protocol for both studies was approved by the University of Arizona Institutional Review Board, and all participants provided informed consent.

## Results

### Study 1.

#### Young vs. older group differences.

This lifespan sample (18 to 89 y) consisted of 1,974 young, 733 middle-age, and 1,140 older adults. Beyond overall frequency of occurrence, we focused on 5 critical dimensions of autobiographical thought assessed in MW: 1) emotional valence (Affective Content), 2) spatiotemporal specificity (Episodic Specificity), 3) clarity and vividness (Vividness), 4) Self Focus and 5) Social Orientation. Temporal orientation and Task-Relatedness were also analyzed in follow-up analyses.

A linear regression model predicting the proportion of autobiographical thoughts from age group revealed that older adults experienced significantly lower proportions of autobiographical thoughts compared to younger adults (*M_YA_* = 0.40, *M_OA_* = 0.26, ***β*** = −0.101, ***SE*** = 0.008, ***R^2^*** = 0.043, ***P*** < 0.001).

**Fig. 1.**
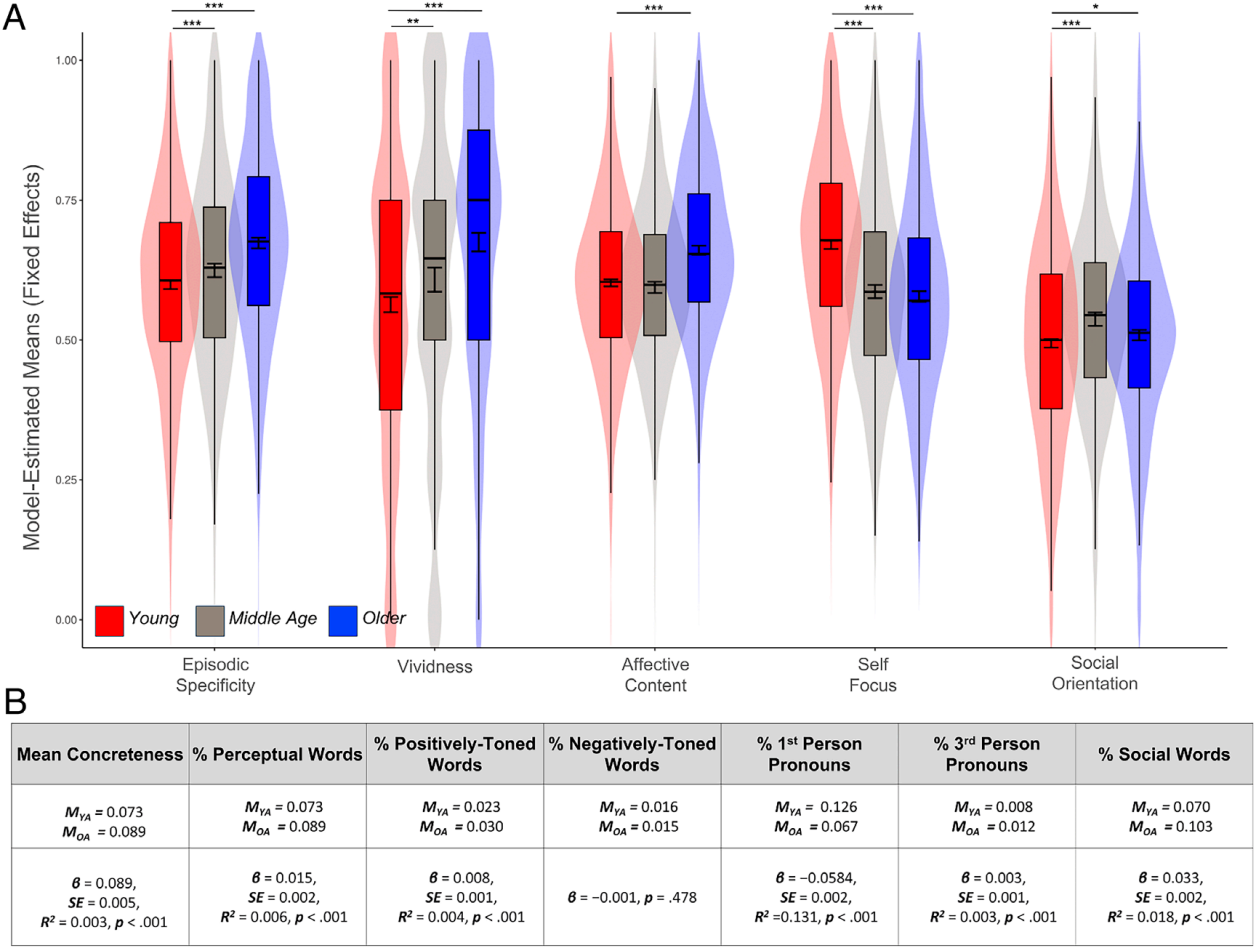
(*A*) Model-estimated means for characteristics of autobiographical thoughts in Young, Middle-aged, and Older Adults. (*B*) Model estimates for linguistic features in Ref. 7 (concreteness) and Ref. 8 (all other categories) for Young and Older Adults. *P < 0.05, ***P < 0.001.

Next, we used stepwise linear mixed-effects modeling to examine the effect of age group on phenomenological characteristics of interest. Compared to younger adults, older adults rated their autobiographical thoughts more episodically specific (***β*** = 0.075, ***SE*** = 0.006, ***R^2^*** = 0.014, ***P*** < 0.001), more vivid (***β*** = 0.112, ***SE*** = 0.011, ***R^2^*** = 0.025, ***P*** < 0.001), more positive in affective content (***β*** = 0.058, ***SE*** = 0.005, ***R^2^*** = 0.017, ***P*** < 0.001), less self-focused (***β*** = −0.092, ***SE*** = 0.006, ***R^2^*** = 0.022, ***P*** < 0.001) and more socially-oriented (***β*** = 0.015, ***SE*** = 0.006, ***R^2^*** = 0.002, ***P*** = 0.015) (Fig. 1*A*).

According to temporal construal theory (9), these age differences would be expected if older adults exhibited a greater proportion of present-focused or temporally proximal autobiographical thoughts than younger adults. However, older adults did not differ from young adults on present-focus (*P* = 0.71) and reported less frequent temporally proximal autobiographical thoughts (*M_YA_* = 0.23, *M_OA_* = 0.20, ***β*** = 0.023, ***SE****: 0.008, P* = 0.02). Despite these group differences, older adults showed effects in line with temporal construal theory, namely, that thoughts about one’s distant past or future were rated less specific than thoughts about one’s more proximal past or future (***β*** = −0.039, ***SE****: 0.008,* ***R^2^*** = 0.019, *M_proximal_* = 0.74, *M_distal_* =0.70, *P* < 0.001), further validating the EMA approach across age groups. Moreover, all age effects, except Social Orientation (*p = 0.057),* remained significant when restricting our data to autobiographical memories only (ps < 0.001 to 0.010) and when controlling for task relatedness (ps < 0.001 to 0.011). More details on OSF (https://osf.io/qa79e.).

Next, participants’ free-response thought descriptions were analyzed by referencing words to semantic categories (7, 8) comparable to MW variables of interest, in line with prior work in the autobiographical thinking literature (10). In alignment with the self-report findings, linear mixed-effects models revealed that older adults’ written descriptions were significantly more concrete than young adults and included a higher proportion of perceptual (e.g., seeing, hearing, feeling) and positively toned words (e.g., good, well, love) (Fig. 1*B*). They did not differ on their use of negatively toned words (e.g., bad, wrong, hate). Older adults also demonstrated less first-person language usage (e.g., I, me, myself), and more third-person pronoun usage (e.g., he, she, they) and social referencing (e.g., family, friends, people) (Fig. 1*B*).

### Effects in Middle Age.

The phenomenological and linguistic characteristics of middle-aged adults’ autobiographical thoughts generally fell between young and older adult profiles (Fig. 1*A*), with significant differences compared to young and older groups for many metrics. When middle-aged participants were included in models with age as a continuous variable across the adult lifespan, significant linear relationships emerged for all metrics except vividness. Modeling inverse quadratic effects tended to improve model fits. Analyses including middle-aged adults are available on OSF.

### Study 2.

We conducted a second preregistered study (OSF) in 75 young (18 to 34 y) and 142 cognitively normal older adults (60 to 84 y). Participants in Study 2 used Mind Window (MW) and also completed the Autobiographical Interview (AI). Data and procedures were done according to standard protocol (see *SI)* (5).

Confirming our first preregistered hypothesis, older adults reported significantly lower proportions of autobiographical thoughts compared to younger adults (*M_YA_* = 0.40, *M_OA_* = 0.31, ***β*** = −0.095, ***SE*** = 0.030, ***R^2^*** = 0.038, ***P*** = 0.002), replicating findings from Study 1.

Older adults also reported that their autobiographical thoughts were more episodically specific (***β*** = 0.097, ***SE*** = 0.024, ***R^2^*** = 0.026, ***P*** < 0.001), positive (***β*** = 0.058, ***SE*** = 0.017, ***R^2^*** = 0.019, ***P*** < 0.001), and socially oriented (***β*** = 0.051, ***SE*** = 0.024, ***R^2^*** = 0.010, *P* = 0.029) than young adults. There were no significant differences in regard to vividness (***β*** = −0.059, ***P*** = 0.241) or *self-focus* (***β*** = −0.045, *P* = 0.073).

As specified in our preregistered hypothesis (H5), subsequent analyses focused on autobiographical memories and future thoughts, which engage similar constructive processes (2). As expected, older adults provided a smaller proportion of internal (episodic)/total details on the AI [*M_YA_* = 0.663, *M_OA_* =0.550, *t*(123.85) = 6.25, *P* <0.001]. Internal detail proportion did not predict MW episodic specificity (***β*** = –0.16, ***P*** = 0.477) and, contrary to our hypothesis, no age x internal detail interaction emerged (***β*** = 0.22, ***P*** = 0.238).

## Discussion

Our findings offer an alternative perspective on theories of cognitive aging. Contrary to global decline-oriented views, Study 1 showed age-related increases in episodic specificity across self-report and linguistic indices. This pattern persisted in Study 2, where increased episodic specificity in Mind Window paralleled decreased specificity via the Autobiographical Interview, a commonly used laboratory task.

Age-related episodic specificity increases are not easily explained by self-report biases or metacognitive deficits as behavioral patterns were supported by linguistic analysis and showed consistency with other findings in the literature, namely temporal construal theory (9) and the commonly observed age-related positivity effect (11). Instead, we suggest that divergences from lab-based findings may reflect contextual influences in aging (12), such as shifts in narrative goals in social settings (3), discrepancies in background knowledge between older participants and experimenters, stereotype threat (13), and the absence of personal environmental retrieval cues (14, 15). Importantly, our inclusion of middle-aged adults offers a more continuous view of lifespan trajectories, revealing that these effects are not limited to extremes of age, underscoring the value of age as a continuum rather than dichotomous variable.

Despite age-related decreases in the overall frequency of everyday autobiographical thoughts, our phenomenological findings pertaining to such thoughts fit with growing evidence that older adults perform as well or better in spontaneous or naturalistic memory contexts. Prior studies have shown no age deficits, and even advantages, in involuntary memory (16), direct retrieval (17), and naturalistically assessed prospective memory (4). Together, this literature supports the idea that lab-based cognitive decline may not be as generalizable as once thought (18, 19), prompting methods that capture a fuller range of factors underlying this phenomenon.

Additionally, our results align with work suggesting that emotional and motivational factors shape autobiographical thinking across adulthood. Consistent with theories of socioemotional selectivity and the “positivity bias” (11), older adults showed increases in positive and socially relevant content. This invites the possibility that affective biases in advanced age may enhance episodic richness by focusing attention on meaningful experiences thus promoting rich, detailed memory. In this light, positivity and specificity may be intertwined processes in older age, particularly when engaging in unprompted, self-generated thought. This challenges the assumption that aging entails a universal loss of episodic detail and suggests that, in day-to-day contexts, aspects of autobiographical content may be preserved in older age.

The flexibility of EMA can be seen as a limitation and a strength. While users can choose when and where to answer surveys, possibly limiting the extent of contexts sampled, EMA can also enhance ecological validity in a scalable and accessible manner, highlighting their unique value for advancing theories of autobiographical thinking and cognitive aging. Future work could explore how cognitive and socioemotional processes contributing to autobiographical thought interact with neurobiological aging, health, and well-being, ultimately reshaping our understanding of memory and identity across the lifespan.

## Materials and Methods

*Supplementary Methods* appear in the *SI Appendix*.

## Supplementary Material

Appendix 01 (PDF)

## Data Availability

All data used in the present study, with the exception of the raw free response data, are provided on OSF: https://osf.io/qa79e (20), as well as unregistered analyses requested by our reviewers.
