# Supplementary material for: Enhanced episodic specificity and socioemotional content in older adults’ everyday autobiographical thoughts
Source: Proc Natl Acad Sci U S A. 2026 Jan 8;123(2):e2513990123. doi: 10.1073/pnas.2513990123 (PMC12799099; doi:10.1073/pnas.2513990123)
Supplement: Supplementary file 1 — Appendix 01 (PDF) [file pnas.2513990123.sapp.pdf]

Supporting Information for:

Enhanced Episodic Specificity and Socioemotional Content in Older Adults' Everyday Autobiographical Thoughts

Vannia A. Puig Rivera, Eric S. Andrews, Leelu J. Cervantes, Delaney Freveletti, Matt Huentelman, Matthew D. Grilli and Jessica R. Andrews-Hanna

Corresponding Authors: Jessica R. Andrews-Hanna; Vannia A. Puig Rivera
Email: jandrewshanna@arizona.edu; vapuigrivera@arizona.edu

This file includes:

Supporting text
Supporting references

Data, Materials, and Software Availability

All data used in the present study, with the exception of the raw free response data, are provided on OSF: https://osf.io/qa79e

Extended Methods

Procedures Common to Both Studies

The Mind Window App
To collect a wide range of characteristics of everyday autobiographical thoughts across different age groups, we used a novel mobile smartphone application, called Mind Window (MW). MW employs an ecological momentary assessment (EMA) approach designed to capture insights into individuals' momentary thoughts across a range of everyday contexts. MW was developed by the Neuroscience of Emotion and Thought Lab at The University of Arizona and is a freely downloadable app for iOS and Android devices.[1-2] Upon downloading and creating an account, participants provided basic demographic information and completed a comprehensive "Getting to Know You" assessment comprising 83 trait and lifestyle questions. They then selected a preferred time range for receiving daily "check-in" notifications. These check-in notifications were delivered randomly six times per day within the chosen time window, with a minimum gap of 45 minutes between surveys. Notifications prompted participants to answer a ~2-minute survey referencing the mood and thoughts participants experienced just before the check-in. To minimize recall bias, each check-in became inaccessible after 10 minutes.

Each EMA survey included 12 randomly-presented core questions and 2 additional questions from a rotating pool. To ensure that participants understood the questions as intended, questions were explained in more detail in the app through a "question mark" icon next to each question that was accessible to participants from both studies. Additionally, participants in Study 2 were provided with detailed explanations of what each question was asking directly by the experimenter. All participants could also contact our team with questions through a Mind Window support email.

Users were oriented to these icons during the app setup process and could also contact our team with questions through a MW support email. Participants in Study 2 were also personally led through each question by the experimenter to make sure they understood the meaning of the questions.

Questions assessed in the current manuscript included:

Attentional Orientation Question:

"In the moments just before the notification, where was the focus of your attention?"

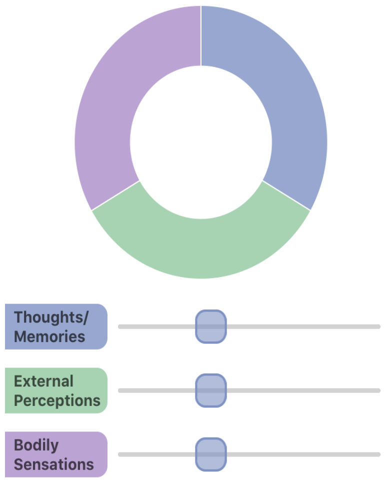

<u>Additional explanation provided to participants:</u>
There are three sliders on this screen. One pertains to 'external' things (sights, sounds, etc.), one to 'internal' things (thoughts, memories, plans, daydreams, etc.), and one to 'bodily' things (pain, physical sensations, etc.). Use the sliders to estimate where your attention was oriented by dividing among these categories. It's best to set the largest value first, then the lowest. The middle value will set itself.

For each entry, participants chose precisely *how much* of their attention was directed to *each of the three* attentional categories (i.e., Thoughts/Memories, External Perception, Bodily Sensations) by sliding the corresponding button to their desired response. Responses were coded for each category from 0 – 1, where all 3 responses sum to 1.0. In other words, the responses are relative to one another.

Episodic Specificity Question:
"In the moments just before the notification, how specific to a particular time and/or place were your thoughts?"

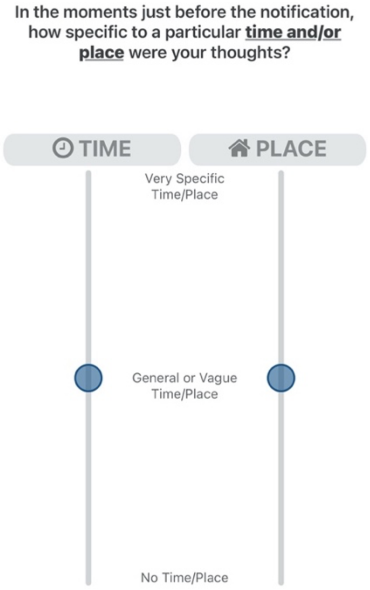

Additional explanation provided to participants:
"Use these two sliders to describe whether your thoughts, at the time of the notification, were about a very specific time and/or a very specific place that you might have been (or might be in the future). For example, thinking about your eighth birthday at your grandmother's house would be very specific on both scales, while thinking that you should be exercising more often may be very non-specific to a particular time and place."

Participants chose precisely *how specific or general* their thought was directed to a certain time or place by sliding the slider (responses ranged from 0-1). Temporal and spatial specificity were assessed in separate questions. Episodic Specificity was conceptualized as the average of these two responses. These variables capture the degree of spatiotemporal specificity within participants' thoughts.

Affective Content Question:
"In the moments just before the notification, how positive or negative were your thoughts?"

Additional explanation provided to participants:
"Use the slider on this screen to indicate how unpleasant, negative, unhappy, unsatisfying, pessimistic, melancholic, or despairing your thoughts were at the time of the notification or how pleasant, positive, happy, satisfying or optimistic they were."

Participants answered using a sliding scale with the anchors below, recorded from 0 to 1 (Neutral = 0.5).

(1)- Very positive
- Somewhat positive
- Neutral
- Somewhat negative
(0)- Very negative

Self-Focus and Social Orientation Questions:
"In the moments just before the notification, to what degree were your thoughts about you? To what degree were they about other people?"

Additional explanation provided to participants:
"Use the graphics on this screen to indicate how much you were thinking about yourself, at the time of the notification, and how much you were thinking about other people. Fill both the individual figure and the group figure to the level that seems appropriate. It's ok to have them both filled (or both empty) – that just suggests that your thoughts were equally, and very strongly (or weakly) focused on both yourself and others.

Participants moved the slider separately for self-focus (icon on the left) and social orientation (icons on the right). Responses for each question range from 0-1, from bottom (unfilled) to top (filled).

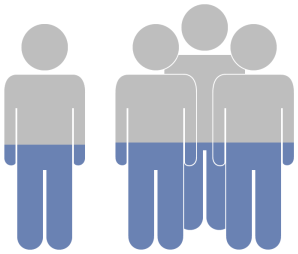

Temporal Orientation
"In the moments just before the notification, in what timeframe where your thoughts ***primarily*** *focused?*

Participants selected one of the discrete options below:

- No Time at All
- More than 2 weeks ago
- Less than 2 weeks ago
- Now (within current 5 minutes)
- Less than 2 weeks from now
- More than 2 weeks from now

Vividness Question (a rotating question):
"In the moments before the notification, overall, how vivid were your thoughts in your "mind's eye?""

Additional explanation provided to participants:
"Use the buttons below to select the description that best describes how clear and detailed images in your mind were at the time of the notification."

Participants selected one of 5 discrete options below, built from response options drawn from the Visual Vividness Inventory Questionnaire (VVIQ)[3].

(5)- Perfectly clear and vivid as normal vision
- Clear and reasonably vivid
- Moderately clear and vivid
- Vague and dim
(1)- No image at all (you only "know" you are thinking of something)

Task-Relatedness Question:
"In the moments just before the notification, to what degree were your thoughts/attention focused on your primary task or activity at hand".

Participants answered using a sliding scale with the anchors below, recorded from 0 to 1.

(1)- Very much so
- Quite a bit
- Somewhat

- A Little
(0)- Not at all

Additional explanation provided to participants:
Use the sliders to indicate how much of your thoughts, at the time of the notification, were focused on what you were actively trying to do at the time. Move the slider to "Very much so" if you were completely focused on the primary "task" (e.g., working, doing the dishes, watching TV, jogging, etc.).

Free Response Description:
Following each set of self-report questions, participants were invited to describe their thoughts in a free response question. Participants had up to 1,000 words:

"Please use this space to describe what you were thinking about in the moments just before the notification (this is optional, but the more you write adds to the value of the statistics we can show you)."

Defining Autobiographical Thoughts
Autobiographical thoughts were defined as surveys focused at least 0.5 (50%) on thoughts/memories (assessed on the *Attentional Orientation* question) and at least 0.10 on self-focus. Each participant's overall proportion of autobiographical thoughts was calculated as the number of surveys characterized as pertaining to an autobiographical thought divided by the number of total surveys completed.

Temporal Construal Analyses
For our analyses related to temporal construal, "Proximal" refers to thoughts characterized as pertaining to events <2 weeks from now/ago and "Distal" to >2 weeks from now/ago. "Present" (within 5 minutes) was excluded from analysis.

Study 1
Sample and Data Collection procedures
The full experimental protocol was approved by the University of Arizona Institutional Review Board (IRB) and all participants provided informed consent. Data were collected from April 2020 to June 2024, encompassing responses from 6,018 individuals recruited through various channels, including convenience sampling (through Google and iOS app stores), recruitment via the University of Arizona's SONA undergraduate psychology student pool, and through partnerships with collaborative data registries including the Alzheimer's Prevention Registry, GeneMatch and MindCrowd, developed by the Translational Genomics Research Institute. Individuals who did not complete the account setup or contribute at least 10 Ecological Assessment (EMA) surveys (n=2,169) were excluded.

Within the sample, eight participants did not have the expected number of responses for the *Timeframe* measure (due to a bug in one version of the Mind Window app) so they were excluded. Due to an application error early in the data collection process, some participants received more than six check-in prompts per day. For that reason, 21 participants had more than the expected number of samples.

The final sample consisted of 3,847 participants (age range: 18-89 years, *M*age = 39.8). The sample consisted of predominantly cisgender women (70%), 28% cisgender men and 2% identifying as non-binary, transgender, or self-described.

Of the 3,847 participants, 66.9% (n=2573) identified as Non-Hispanic White. 13.7% (n=528) identified as Latinx, Hispanic, or Hispanic American, followed by those identifying as Multiracial/Other, totaling n=314 individuals (8.2%). Asian or Asian American participants accounted for n=214 individuals (5.6%). Remaining proportions of the sample included Black or African American participants (2.6%;

n=101). Native American, Alaska Native, First Nations, or Indigenous participants (n=35; 0.9%). The smallest group was Native Hawaiian or Other Pacific Islander, with n=12 individuals (0.3%). Seventy individuals (1.8%) preferred not to answer.

To be included in the analysis of Study 1, participants were required to complete at least 10 surveys in a two-week time period, but participants could use the MW app for as long as they wished. Participants included in Study 1 completed at least one survey within the app, on average, 13.3 days.

In Study 1, participants, on average, completed thought descriptions during 68.2% of all surveys started. Of 3,847 participants, 3,534 (91.9%) provided at least one written thought description.

Statistical Analyses

Proportion of Autobiographical Thoughts

To examine age differences in the proportion of autobiographical thoughts out of all surveys answered, we applied a linear regression with proportion of autobiographical thoughts as the outcome variable and age group as the predictor.

Phenomenological Ratings

To examine the effect of age group on our characteristics of interest obtained via the Mind Window (MW) app, we used a hierarchical linear mixed-effects modeling (LMM) approach using the **lme4**[4] package in **R**. This approach is appropriate for analyzing data with nested structures, such as repeated observations within individuals.

For all models, the step-building process began with a null model, including only a random intercept for participants (P.ID) to account for individual variability and to estimate the baseline Intraclass Correlation Coefficient (ICC). The ICC indicated the proportion of variance in the dependent variable attributable to individual differences.

Subsequently, fixed effects were added in a stepwise manner, with each variable of interest set as the dependent variable. All models included a random intercept (P.ID) to account for the nested structure of the data (i.e., repeated observations within participants). After each addition, model parameters were examined using and confidence intervals were computed. The models were fitted using the Restricted Maximum Likelihood (REML) method, formally comparing the nested models to determine if the inclusion of additional fixed effects significantly improved model fit. This iterative process allowed for the identification of the most parsimonious and best-fitting model that explained the variance in each variable.

Task-Relatedness Analyses

Autobiographical thoughts could vary in their degree of task-relatedness. For example, writing in one's diary about overcoming life challenges, writing down patient notes while recalling similar cases, or cleaning out a closet and reflecting about events associated with items would all constitute autobiographical thoughts that reflect some degree of task-relatedness. As older adults reported significantly higher task-relatedness than younger adults (β = 0.10 *SE* = 0.006, p< .001), we re-ran all primary analyses controlling for task-relatedness to determine whether age effects on autobiographical thought characteristics (episodic specificity, vividness, affective content, self-focus, social orientation) persisted after accounting for this variable. All significant age effects remained, even when controlling for task-relatedness. These results demonstrate that age-related differences in autobiographical thought within our sample are not confounds of differential task engagement.

Linguistic Analysis

To analyze the linguistic dimensions of participants' everyday thoughts, we began by preprocessing raw text entries using the following steps: (1) removal of entries with fewer than eight words to ensure individual entries had sufficient linguistic content, (2) standardization of text (e.g., lowercasing, removal of extraneous punctuation), and (3) exclusion of non-English responses where applicable. Tokenization and word count normalization were then applied to facilitate comparison across entries of varying lengths.

LIWC-2022. Linguistic Inquiry Word Count (LIWC-22)[5] software was used to extract linguistic features for 1) *perceptions* (e.g., seeing, hearing, feeling), 3) *positive and negative tone* (e.g., good, well, love/bad, wrong, hate), 4) *first and third-person pronoun usage* (e.g., I, me, myself/he, she, they), and *social references* (e.g., family, friends, people) as these categories best reflected the self-reported questions of interest. Proportions of words in each category were extracted for analysis. To investigate age-related patterns in language use, we applied the same hierarchical LMM approach described above.

For all models involving LIWC analyses, the step building process began with a null model, including only a random intercept for participants (P.ID) to account for individual variability and to estimate the baseline Intraclass Correlation Coefficient (ICC). The ICC indicated the proportion of variance in the dependent variable attributable to individual differences.

Subsequently, fixed effects were added in a stepwise manner, with each variable of interest set as the dependent variable. All models included a random intercept (P.ID) to account for the nested structure of the data (i.e., repeated observations within participants). After each addition, model parameters were examined using Likelihood Ratio Tests (LRT) and confidence intervals were computed. The models were fitted using the Restricted Maximum Likelihood (REML) method, formally comparing the nested models to determine if the inclusion of additional fixed effects significantly improved model fit. This iterative process allowed for the identification of the most parsimonious and best-fitting model that explained the variance in each variable.

Concreteness. To analyze the linguistic dimension of *concreteness* of participants' free-text thought descriptions, we referenced free response thought descriptions to a concreteness dictionary of 40,000 words.[6] To prepare the text for analysis, all entries were tokenized into individual words, which were then converted to lowercase. These tokens were subsequently joined with the cleaned concreteness dictionary, allowing for the assignment of a concreteness score to each word. Words not present in the dictionary were implicitly excluded from the concreteness calculation. Individual words were categorized as "Abstract" (concreteness score < 3), "Concrete" (concreteness score > 4), or "Neutral" based on their assigned scores. For each participant, an average concreteness score was then computed across all their available words.

Linear Mixed Models (LMMs) were initially employed to investigate the relationship between age group and average concreteness, accommodating the nested structure of the data (multiple words per participant). A null model, including only a random intercept for participant ID, was initially established to quantify baseline variance and compute the Intraclass Correlation Coefficient (ICC). A subsequent LMM then incorporated age group as a fixed effect, with model parameters estimated using Restricted Maximum Likelihood (REML).

However, when the null LMM was run to assess the intraclass correlation coefficient (ICC) for concreteness, which represents the proportion of variance in concreteness explained by differences between participants, the ICC for concreteness was found to be very low (ICC=0.007). Given this negligible amount of variance attributed to individual participants, we determined that a multi-level approach was not necessary. Consequently, we proceeded with standard linear regression models to examine the effect of age group on average concreteness. A linear regression model was fitted to predict

concreteness based on age group. The difference in estimates for both models was negligible (in both cases $\beta \approx 0.89$)

Study 2 (Preregistered, https://osf.io/qa79e)
Sample and Data Collection procedures
The full experimental protocol was approved by the University of Arizona Institutional Review Board (IRB) and all participants provided informed consent. Study 2 included data from two combined samples collected by our research laboratory, of which both participants samples were independent from Study 1. In both samples, the exclusion criteria were current drug abuse, major psychiatric disease, except for mild-to-moderate depression, history of moderate to severe head trauma, neurodevelopmental disorder, or not being fluent in English. Vulnerable populations were also not enrolled in this research. Participants were recruited via the University of Arizona Psychology website, departmental listservs, social media advertisements and via referral through participation in other studies. Participants received either cash compensation or gift cards via email.

The full sample for Study 2 (n=217) includes 75 young (age range = 18-34, *M*age = 24.1, 54 cisgender women, 2 non-binary) and 142 older adults (age range = 60-84, *M*age = 69.4, 107 cisgender women) between the ages of 18 – 84. The decision to harmonize both study samples was made because the experimental procedures of main interest for this work were the same in both studies, with the exception that Study A completed the Autobiographical Interview via Zoom, and Study B completed the Autobiographical Interview in person. Otherwise, participants engaged with the Mind Window app in a similar manner. In terms of timeline, Study A was collected between 2020-2021 and includes 93 participants. Study B began collection in 2021 and at the time of manuscript revision, continues in the process of data collection. However, we only included participants who had fully completed their experimental procedures at time of data download (January 2025), resulting in 121 participants for Study B.

Across both procedures, older adults completed a neuropsychological and cognitive battery as part of a screening process to include cognitively normal older adults. The battery involved measures of memory (California Verbal Learning Test, Rey Complex Figure Test, language (Boston Naming Task, animal fluency task), attention/executive function (trail making task), and visuospatial abilities (block design, matrix reasoning from Weschler Adult Intelligence Scale). Participants who scored more than two (or more) z-scores below -1 in a single domain (on two tests) or across all domains (on all three tests) were excluded from participation.

Of the 217 participants, the majority, 77% (n=167; young=39) identified as Non-Hispanic White. Thirteen percent (n=29, young=21) identified as Latinx, Hispanic, or Hispanic American. Other racial identifications included 4.1% (n=9; young=9) Asian or Asian American, 1.8% (n=4; young=1) Black or African American, and 1.8% (n=4; young=2) Multiracial/Other. A small percentage of participants identified as Native American, Alaska Native, First Nations, or Indigenous (0.9%, n=2; young=2). Two (0.9%; young = 0) participants preferred not to answer.

In Study 2, a complete study involved use of the MW app across 10–12 days. However, participants could still choose to use the app beyond study completion. For analysis in Study 2, we also included any data beyond the 10-12 study period to increase statistical power, and as in Study 1, we used linear mixed effects analyses to account for a different number of surveys across participants. Participants included in Study 2 completed at least one survey within the app, on average, 13.6 days.

In Study 2, participants on average completed thought descriptions during 73.6% of all surveys started. Of 217 participants, 205 (94.4%) provided at least one written thought description.

Autobiographical Interview (AI)[7]

Participants in Study 2 were asked to recall five distinct events from their lives, each lasting less than 24 hours, following the AI free recall and probing instructions[7]. Participants described these autobiographical memories aloud to an experimenter and narratives were transcribed and scored using the AI scoring protocol, which distinguishes between internal (episodic, event-specific) details (e.g., perceptual, temporal, emotional) and external details (e.g., semantic knowledge, repetitions, secondary events). The current study computed a proportion of internal/total details as our primary metric of internal detail. Each narrative was scored by two raters who were trained in the Autobiographical Interview. Each rater achieved high interrater reliability for internal and external details (i.e., ICC > 0.90) with expert scorers on a standard memory dataset used for training. The two raters' scores were averaged for each participant, creating a more reliable estimate of their use of detail types.

Of the 217 participants, 3 experienced audio recording issues, so they were removed from the analysis pertaining to comparisons between Mind Window and the Autobiographical Interview (see below for additional detail).

AI data for a subset of participants included in the present study has been analyzed as part of previous publications, but in each case, to explore different overarching questions than those in the present manuscript. See OSF link for details.

Statistical Analyses

Mind Window.

To examine age differences in the proportion of autobiographical thoughts out of all surveys answered, we applied a linear regression with proportion of autobiographical thoughts as the outcome variable, gender and study as covariates, and age group as the predictor.

To examine the effect of age group on our characteristics of interest obtained via the Mind Window (MW) app, we used the same stepwise linear mixed-effects modeling (LMM) approach using the **lme4** package in **R** as in Study 1.

To examine the task-by-age interaction between episodic specificity (MW) and proportion of internal / total details (AI), we used a linear mixed effects model with MW as the outcome, AI as the predictor, age*internal proportion as the interaction term, gender, and sample (i.e., Study A/Study B) as covariates, and "Participants" as a random intercept to account for the different number of surveys completed per individual. The variable AI was a calculation of participants' internal-detail score relative to their total-detail score on the Autobiographical Interview, capturing the proportion of internal details within participants memories.

Comparisons between Mind Window and the Autobiographical Interview.

To draw comparisons between episodic specificity (MW) and internal detail (AI) for each age group as outlined by pre-registered hypotheses (i.e., Hypothesis 5), we first made sure we were analyzing comparable data. Since the AI procedures completed by our 214 participants were meant to capture ***memories,*** and Mind Window captures a large variety of thought types, we chose to focus our MW analysis on autobiographical thoughts that were rated as pertaining to past (i.e., memories) or future (i.e., prospections). We chose to include both memories and prospections to 1) increase statistical power (only 14% of participants' surveys were characterized as pertaining specifically to autobiographical memories) and 2) because of the extensive body of research that has indicated strong behavioral and neurophysiological similarities between remembering the past and imagining the future.[5] This literature has led to the understanding that simulations of specific episodes from the past and future draw on similar information and rely on similar underlying processes. The *constructive episodic simulation hypothesis* suggests that episodic memory supports the construction of future events by extracting and recombining stored information into novel simulations.[8-10]

Furthermore, to ensure we captured an average representation of memory characteristics, we implemented a 4-memory/future thought minimum for this subset of data. In other words, participants were included in this analysis only if they had provided **at least** 4 memories or 4 future thoughts in their MW data, reducing our sample to N = 94 participants. The reasoning behind selecting a minimum of 4 versus a higher number was to conserve the power to reach a detectable effect size.

To investigate relationships between internal proportion (AI) and episodic specificity, a Linear Mixed Model (LMM) was constructed using the same stepwise approach (**lme4** package in **R)** as described above, with episodic specificity (MW) set as the dependent variable. The model included internal proportion (AI), age groups, gender and study as fixed effects. A key aspect of this model was the inclusion of an interaction term between internal proportion and age group to explore whether the relationship between internal proportion (AI) and episodic specificity (MW) differed across age groups. To account for the hierarchical structure of the data, "Participant" was included as a random intercept, allowing for variability in baseline episodic specificity across individual participants. The analysis was conducted on a reduced dataset which contained 3,907 observations from 94 unique participants.

Supporting References:

1. E. S. Andrews, M. D. Grilli, S. Bethard, M. R. Mehl, J. J. Allen, J. Arch, & J. R. Andrews-Hanna. Patterns of Everyday Thought as Correlates of Psychological Well-Being and Possible Targets for Personalized Mental Health Treatment. *PsyArXiv.* (2024)
2. Q. Raffaelli, E. S. Andrews, C. C. Cegavske, F. F. Abraham, J. O. Edgin, & J. R. Andrews-Hanna. Dreams share phenomenological similarities with task-unrelated thoughts and relate to variation in trait rumination and COVID-19 concern. *Scientific reports*, *13*(1). (2023)
3. D. F. Marks. Vividness of Visual Imagery Questionnaire (VVIQ) [Database record]. *PsycTESTS.*
4. D. Bates, M. Maechler, B. Bolker, S. Walker. Fitting linear mixed-effects models using lme4. *Journal of Statistical Software* 67, 1–48 (2015).
5. R. L. Boyd, A. Ashokkumar, S. Seraj, J. W. Pennebaker. The development and psychometric properties of LIWC-22. *University of Texas at Austin 10*, 1–47 (2022).
6. M. Brysbaert, A. B. Warriner, V. Kuperman. Concreteness ratings for 40 thousand generally known English word lemmas. *Behavior Research Methods* 46, 904–911 (2014).
7. B. Levine, E. Svoboda, J. F. Hay, G. Winocur, M. Moscovitch. Aging and autobiographical memory: Dissociating episodic from semantic retrieval. *Psychology and Aging* 17, 677–689 (2002).
8. R. G. Benoit, D. L. Schacter. Specifying the core network supporting episodic simulation and episodic memory by activation likelihood estimation. *Neuropsychologia* 75, 450–457 (2015).
9. D. R. Addis, R. P. Roberts, D. L. Schacter. Age-related neural changes in autobiographical remembering and imagining. *Neuropsychologia* 49, 3656–3669 (2011).
10. D. L. Schacter, D. R. Addis. On the constructive episodic simulation of past and future events. *Behavioral and Brain Sciences* 30, 331–332.(2007).
